# Impact of substructure radiation dose on health-related quality of life in children with brain tumors: a Pediatric Proton/Photon Consortium Registry (PPCR) study

**DOI:** 10.1007/s11060-025-05211-w

**Published:** 2025-09-09

**Authors:** Mikaela Doig, Jae Lee, Young Kwok, Iain MacEwan, Suzanne Wolden, Keith Allison, Sara Dennehy, Benjamin Bajaj, Michala Short, Peter Gorayski, Eva Bezak, Torunn I. Yock

**Affiliations:** 1 Allied Health and Human Performance Academic Unit, University of South Australia, GPO Box 2471, Adelaide, SA 5001 Australia; 2 Department of Radiation Oncology, Royal Adelaide Hospital, Adelaide, Australia; 3 Australian Bragg Centre for Proton Therapy and Research, Adelaide, Australia; 4 Department of Radiation Oncology, ProCure Proton Therapy Center, Somerset, USA; 5 Department of Radiation Oncology, Maryland Proton Treatment Center, University of Maryland, Baltimore, USA; 6 Department of Radiation Oncology, California Protons Cancer Therapy Center, San Diego, USA; 7 Department of Radiation Oncology, New York Proton Center, New York, USA; 8 Department of Radiation Oncology, Massachusetts General Hospital, Boston, USA; 9 Harvard Medical School, Harvard University, Boston, USA

**Keywords:** Pediatric oncology, Proton radiotherapy, Health-related quality of life, Brain cancer, Brain substructures

## Abstract

**Purpose:**

Cranial irradiation is associated with health-related quality of life (HRQoL) deficits in childhood cancer survivors. We investigated the relationship between radiation dose to brain substructures and HRQoL in children with brain tumors treated with proton beam therapy (PBT).

**Methods:**

Data were obtained from children in the Pediatric Proton/Photon Consortium Registry who received PBT for primary brain tumors between 2015 and 2021. HRQoL was assessed using PedsQL Generic Core questionnaires during the first week of PBT and annually thereafter. Standardized substructure segmentations and dosimetry data were correlated with parent-proxy reported HRQoL scores using Pearson correlations.

**Results:**

Seventy-six patients were included, with median age 8.9 years (range 1.2–16.5) at diagnosis. Median follow-up was 5.0 years post-PBT and median prescribed dose was 54Gy_RBE_. HRQoL scores were lower than normative population values during PBT, particularly in those who received craniospinal irradiation (CSI) (*p* < 0.05). Across the total cohort, higher mean doses to the whole brain, supratentorial brain, corpus callosum, left hippocampus, hypothalamus, optic chiasm, pituitary and thalamus correlated with worse HRQoL (*r*= -0.24 to -0.39), *p* < 0.05). In the CSI subgroup (*n* = 17), moderate-to-strong associations between dose to these structures and physical functioning (*r*= -0.50 to -0.79, *p* < 0.05) were observed. In children who received focal PBT (*n* = 59), weaker associations were observed between dose to the hypothalamus and pituitary, and HRQoL (*r* = -0.28 to -0.36, *p* < 0.05).

**Conclusion:**

Higher radiation doses to specific brain substructures were associated with poorer HRQoL outcomes after PBT. Minimizing dose to these areas during treatment planning may help preserve HRQoL in survivors.

**Supplementary Information:**

The online version contains supplementary material available at 10.1007/s11060-025-05211-w.

## Introduction

Survivors of childhood brain tumors are at risk of impaired health-related quality of life (HRQoL) [1]. Whilst radiotherapy (RT) is a critical component of care for many patients, RT has been associated with adverse events and chronic health conditions that can impact HRQoL of survivors [2–5].

The negative sequalae of RT are related to the dose, fractionation and volume of normal tissues exposed [6]. Proton beam therapy (PBT) is increasingly used for the treatment of pediatric brain tumors due to the superior sparing of normal tissues, therefore reducing the likelihood of adverse effects, when compared to photon RT [7]. An expanding evidence base demonstrates that increasing RT dose to critical structures can impact key endpoints for survivors of childhood brain tumors, including neurocognition, brain necrosis, cerebrovascular events, ototoxicity, visual toxicity and endocrine dysfunction [8–12]. While adverse effects of treatment are associated with poorer HRQoL outcomes [13], the presence of toxicity alone does not fully capture or characterize the impact of cancer treatment on a child’s HRQoL. Therefore, assessment of HRQoL using patient-reported outcome measures (PROMs) is essential to gain direct insights into the effects of cancer and its treatment.

Studies describing HRQoL outcomes in children with cancer often lack standardized reporting of RT variables, including dose, modality, field extent, or the use of craniospinal irradiation (CSI) [14]. This inconsistency hinders comprehensive analysis and comparison across studies. Previous studies have analyzed the association between dose to brain structures and PROMs in adults with brain tumors [15], and the relationship between estimated dose to brain regions and HRQoL outcomes in long-term adult survivors of childhood cancer [16]. However, the association between RT dose to brain structures and HRQoL in children with a brain tumor remains unknown. Therefore, this study aimed to identify the relationship between dose to brain substructures and HRQoL in children treated with PBT for a primary brain tumor.

## Methods and materials

This study analyzed prospectively collected data by the Pediatric Proton/Photon Consortium Registry (PPCR), a comprehensive multi-institutional registry of childhood cancer patients treated with RT. Informed consent was obtained for all patients prior to enrollment. Institutional Review Board (IRB) approval for the PPCR was obtained from the Dana Farber Cancer Institute (Protocol 12–103, NCT01696721) and subsequently by all participating study sites. This analysis was approved by the University of South Australia’s Human Research Ethics Committee (ID: 204807).

### Study population

The data collection methodology and processes of PPCR have been described previously [17]. PPCR was queried to identify all children diagnosed with a primary brain tumor who were under 22 years old at the time of PBT and had HRQoL data available from during and after PBT. Participants with at least two years of follow-up HRQoL data were included. Participants who received a partial course of RT, those who received photon RT and participants where the cumulative plan could not be retrieved were excluded from analysis. Participants received PBT at the enrolling PPCR centers of Massachusetts General Hospital, ProCure Somerset, Maryland Proton Therapy Center, California Protons Cancer Therapy Center and New York Proton Center.

### Procedure

Demographic, treatment, imaging, planning and outcome data from eligible participants were acquired and reviewed. Data transfer, anonymization, storage and management of RT dose, planning CT and structure set files were facilitated using MIMcloud^®^ (MIM Software Inc., Cleveland, USA). Demographic, treatment, HRQoL and outcome data were extracted from the registry REDCap database [18, 19]. Median household income calculated by residential zip code was used as an estimate of socioeconomic status [20]. All data was managed in a deidentified format.

Contours of intracranial substructures were delineated on the treated plan usually by the treating physician or anatomist and were reviewed for compliance with published guidelines [21–24]. Modifications or missing structures were contoured by a trained dosimetrist (MD) and verified by a pediatric radiation oncologist (TY). Radiation dosimetry were reviewed for all patients. Dosimetry data were derived from clinically delivered treatment plans, with no retrospective optimization of substructures to ensure outcomes reflected the clinically delivered plans. Patients were not treated with advanced avoidance techniques such as whole brain RT with hippocampal or hypothalamic-pituitary axis sparing. Mean and maximum dose for each structure were calculated and included for analysis. Dose is described in Gy_RBE_ using the RBE value of 1.1.

### Patient-reported outcome measure

Pediatric Quality of Life Inventory (PedsQL) 4.0 Generic Core Scales and the PedsQL Infant Scales were used to collect child self-reported (CSR) and parent-proxy reported (PPR) outcomes [25, 26]. PedsQL is a widely used generic measure of HRQoL with validation in both healthy and cancer cohorts [27]. PedsQL Generic Core is a validated 23-item questionnaire that assesses physical, emotional, social and school domains [25]. It includes age-appropriate questionnaire formats, with child self-reporting from age 5 and parent-proxy reporting from age 2. The PedsQL Infant Scales were used for children aged 1 to 24 months. The Infant Scales contain five domains including physical functioning, physical symptoms, emotional functioning, social functioning, and cognitive functioning [26]. The Infant Scales are a parent-proxy questionnaire with the same format, instructions, response scale, and scoring methods as the Generic Core Scales. Items are reverse scored and linearly transformed to a 0-100 scale, with higher scores indicating better HRQoL. Registry participants were administered the PROM using the paper-based form or electronically via REDCap during the first week of PBT, at the end of PBT, and annually thereafter.

### Statistical analysis

Descriptive statistics were used to summarize patient demographic and clinical characteristics. Patient characteristics included age, sex, tumor location, surgical extent, chemotherapy use, hydrocephalus, use of CSI, median income, and ECOG performance status.

PedsQL summary scores were summarized with mean and standard deviation. The study population’s HRQoL scores were compared to that of a cohort of healthy children [27] using the two-sample t-test. Yearly changes in HRQoL were assessed using a linear mixed model with patient as a random effect to account for individual variability in baseline HRQoL and longitudinal trajectories over time. The association between HRQoL scores at baseline and follow-up with demographic and treatment characteristics were evaluated using the independent two-sample t-test. The paired t-test was used to evaluate changes in HRQoL over time within patient subgroups. We assessed the relationship between radiation dose to brain substructures and HRQoL using Pearson correlation coefficients. Correlations were considered weak if below − 0.4, moderate if between − 0.4 and − 0.7, and strong if −0.7 or above [28, 29]. A total of 270 Pearson correlation tests across the brain substructures, mean and maximum doses, and the three PedsQL summary scores were performed. Since the study was not prospectively designed with high power to test a specific hypothesis at a pre-specified type 1 error, the analysis was considered hypothesis-generating, and no adjustments were made for multiple comparisons [30]. Statistical analyses were performed using R Statistical Software (version 4.3.3, R Foundation for Statistical Computing, Vienna, Austria). All p-values are based on a two-sided hypothesis test with a significance level of 0.05.

## Results

### Patient characteristics

A total of 76 children treated with PBT for a brain tumor between October 2015 and February 2021 met the criteria for inclusion. Median HRQoL follow-up was five years (range, 2–8 years), as calculated from the time of the baseline evaluation to the most recent follow-up PROM. Patient characteristics are presented in Table 1. The median age at diagnosis was 8.9 years (range, 1.2–16.5 years) and 51% of participants were female. A majority (96%) of participants had surgery before PBT and 62% received chemotherapy. The median prescribed dose was 54Gy_RBE_ (29.9-59.4Gy_RBE_).

**Table 1:** Patient characteristics

Characteristic	No CSI dose, *n* = 59^a^	CSI dose, *n* = 17^a^
***Demographic & baseline characteristics***		
**Median age at PBT**,** years (range)**	8.9 (1.2–16.5)	9.1 (4.5–15.1)
**Time between diagnosis and PBT**,** years (range)**	0.3 (0.1–10.5)	0.1 (0.0-5.5)
**HRQoL follow-up**,** years (range)**	5.0 (2.0–8.0)	3.0 (2.0–8.0)
**Sex**		
Female	33 (56%)	6 (35%)
Male	26 (44%)	11 (65%)
**Race/Ethnicity**		
White/Non-Hispanic	43 (88%)	15 (100%)
Other	6 (12%)	0 (0%)
**Median household income**,** $**	95,353 (47,177–240,398)	104,748 (46,358 − 144,191)
**Diagnosis** ^**b**^		
Ependymoma	19 (32%)	0 (0%)
Medulloblastoma	0 (0%)	14 (82%)
Gliomas (including astrocytomas)	14 (24%)	0 (0%)
Germ Cell	8 (14%)	2 (12%)
Craniopharyngioma	8 (14%)	0 (0%)
Other	10 (17%)	1 (6%)
**Tumor location**		
Supratentorial	42 (71%)	3 (18%)
Infratentorial	17 (29%)	14 (82%)
**Baseline clinical characteristics**		
Ventricular-Peritoneal (VP) Shunt	4 (7%)	1 (6%)
Feeding tube	9 (15%)	0 (0%)
Mental health support	2 (3%)	1 (6%)
Hydrocephalus	20 (36%)	13 (81%)
Posterior fossa syndrome	1 (2%)	7 (41%)
**ECOG performance status**		
0	37 (63%)	7 (41%)
1	15 (25%)	5 (29%)
2	3 (5%)	3 (18%)
3	1 (2%)	2 (12%)
Not reported	3 (5%)	0 (0%)
***Treatment characteristics***		
**Treatment modalities**		
Surgery, Chemotherapy + PBT	27 (46%)	17 (100%)
Surgery + PBT	29 (49%)	0 (0%)
Chemotherapy + PBT	3 (5%)	0 (0%)
**Number of surgeries**		
0	3 (5%)	0 (0%)
1	38 (64%)	14 (82%)
2	8 (14%)	2 (12%)
3	7 (12%)	0 (0%)
4+	3 (5%)	1 (6%)
**Surgical extent**		
GTR	34 (63%)	12 (75%)
NTR	4 (7%)	1 (6%)
STR	16 (30%)	3 (19%)
**High dose chemotherapy + Stem cell support**	5 (21%)	2 (13%)
**Antibody/immunotherapy**	3 (6%)	0 (0%)
**Proton therapy modality**		
Passive scatter	29 (49%)	4 (24%)
Pencil beam	24 (41%)	12 (71%)
Pencil beam and passive scatter	6 (10%)	1 (6%)
**Total dose (GyRBE)**	54.0 (29.9–59.4)	54.0 (52.2–54.0)
**CSI dose**		
23.4	-	13 (76%)
27	-	1 (6%)
30.6	-	1 (6%)
36	-	2 (12%)

CSI, craniospinal irradiation, PBT, proton beam therapy, GTR, gross total resection, NTR, near total resection, STR, subtotal resection

^a^Median (Minimum-Maximum); n (%)

^b^Other diagnoses included Embryonal tumors (*n* = 4, 5.3%), Meningioma (*n* = 1, 1.3%), Ganglioglioma (*n* = 1, 1.3%), Neuroepithelial tumors (not otherwise classified) (*n* = 1, 1.3%), ATRT, Atypical Teratoid Rhabdoid Tumor (*n* = 1, 1.3%), Pituitary adenoma/carcinoma (*n* = 1, 1.3%), Choroid Plexus carcinoma (*n* = 1, 1.3%), Rhabdomyosarcoma (*n* = 1, 1.3%)

Seventeen (22%) participants received craniospinal irradiation (CSI) with a median dose of 23.4Gy_RBE_. Results and analyses are stratified based on the presence of CSI due to differences in patient characteristics, substructure doses and HRQoL outcomes. Participants were primarily treated with CSI for medulloblastoma or germ cell tumor. The most common diagnoses among participants who did not receive CSI were ependymoma and astrocytoma. All participants who were treated with CSI received chemotherapy, whilst half (51%) of the non-CSI cohort received chemotherapy. Among the participants who did not receive CSI, seven (12%) received whole ventricular irradiation.

### HRQoL outcomes

Table 2 shows the HRQoL outcomes of the cohort at baseline compared to normative population reference values. HRQoL was lower than the reference values, with statistically significant differences (*p* < 0.05) observed across all CSR and PPR summary scores and domains, except for CSR social functioning. The mean PedsQL total score was 7.2 points lower (*p* = 0.0042) for CSR outcomes and 18.5 points lower (*p* < 0.0001) for PPR outcomes. As presented in Supplemental Table 1, the PPR total score increased by 1.2 points annually following PBT.

**Table 2:** Health-related quality of life outcomes during the first week of PBT and normative reference values

	Score	Healthy Population*		Study Cohort		Difference	p
		n	Mean (SD)	n	Mean (SD)		
Parent	Total Core	717	87.6 (12.3)	76	69.1 (18.8)	−18.5	**< 0.0001**
	Psychosocial	717	86.6 (12.8)	76	71.4 (16.3)	−15.2	**< 0.0001**
	Physical	717	89.3 (16.4)	76	65.6 (27.3)	−23.7	**< 0.0001**
	Emotional	718	82.6 (17.5)	76	68.7 (19.0)	−13.9	**< 0.0001**
	Social	716	91.6 (14.2)	76	79.9 (15.8)	−11.7	**< 0.0001**
	School	611	85.5 (17.6)	43	61.0 (23.7)	−24.5	**< 0.0001**
Child	Total Core	401	83.0 (14.8)	49	75.8 (16.1)	−7.2	**0.0042**
	Psychosocial	399	82.4 (15.5)	48	77.2 (14.1)	−5.2	**0.0201**
	Physical	400	84.4 (17.3)	50	74.5 (23.4)	−9.9	**0.0054**
	Emotional	400	80.9 (15.5)	49	75.4 (18.1)	−5.5	**0.0463**
	Social	399	87.4 (17.2)	48	85.4 (15.7)	−2.0	0.4126
	School	386	78.6 (20.5)	38	67.6 (18.9)	−11.0	**0.0014**

*Healthy population data from Varni et al. 2002. Items are transformed to a 0-100 scale, where a higher score indicates better health-related quality of life. Difference of means assessed using one sample t-test. *p* < 0.05 are shown in bold

### Relationship between HRQoL and clinical variables

Univariate analyses evaluated associations between demographic and treatment variables with HRQoL outcomes. Clinical variables associated with PPR total score are presented in Table 3, while associations with CSR outcomes and PPR physical and psychosocial summary scores are shown in Supplemental Tables 2–6.

**Table 3:** Univariate analysis of parent-proxy reported total core HRQoL and clinical variables

		Baseline		Last follow-up			
Total Core Score	*n* = 76^1^	Mean (SD)	p-value ^2^	Mean (SD)	p-value ^2^	Mean difference (95% CI)	p-value ^3^
**Overall**		69.1 (18.8)		73.3 (22.2)		4.2 (−0.33, 8.73)	0.069
**Age at PBT**			0.207		0.795		
≤ 7 years-old	33 (43.4%)	72.2 (15.6)		74.0 (21.0)		1.84 (−5.43, 9.1)	0.610
> 7 years-old	43 (56.6%)	66.7 (20.8)		72.7 (23.2)		6.01 (0.05, 11.97)	**0.048**
**Sex**			0.897		0.636		
Male	37 (48.7%)	69.4 (17.9)		74.5 (21.5)		5.16 (−0.92, 11.23)	0.094
Female	39 (51.3%)	68.8 (19.8)		72.1 (23.0)		3.29 (−3.66, 10.24)	0.344
**Tumor location**			0.576		0.834		
Supratentorial	45 (59.2%)	70.1 (17.6)		73.7 (22.7)		3.63 (−2.7, 9.97)	0.254
Infratentorial	31 (40.8%)	67.6 (20.6)		72.6 (21.6)		5.02 (−1.64, 11.68)	0.134
**Surgical Extent**			0.508		0.547		
GTR/NTR	51 (72.9%)	70.8 (19.0)		74.4 (20.8)		3.65 (−1.47, 8.77)	0.158
STR	19 (27.1%)	67.4 (19.4)		70.7 (27.1)		3.38 (−7.97, 14.74)	0.539
**Chemotherapy**			**0.034**		0.080		
Chemotherapy	47 (61.8%)	65.5 (18.5)		69.8 (23.4)		4.27 (−2.00, 10.53)	0.177
No chemotherapy	29 (38.2%)	74.9 (18.0)		79.0 (19.0)		4.09 (−2.56, 10.74)	0.218
**Hydrocephalus**			0.218		0.161		
Hydrocephalus	33 (46.5%)	65.8 (19.1)		69.7 (22.9)		3.97 (−3.51, 11.45)	0.287
No hydrocephalus	38 (53.5%)	71.2 (18.0)		77.1 (21.2)		5.90 (−0.19, 11.98)	0.057
**Craniospinal irradiation**			**0.003**		0.342		
CSI dose	17 (22.4%)	57.4 (19.5)		68.8 (18.7)		11.40 (0.83, 21.96)	**0.036**
No CSI dose	59 (77.6%)	72.5 (17.3)		74.6 (23.0)		2.12 (−2.9, 7.15)	0.401
**Median income of zipcode**			0.071		0.351		
≤ $95,000	32 (46.4%)	74.2 (19.0)		74.5 (22.6)		0.25 (−5.83, 6.34)	0.933
>$95,000	37 (53.6%)	66.0 (18.3)		69.4 (22.6)		3.39 (−3.43, 10.21)	0.321
**ECOG**			**0.047**		0.591		
ECOG 0	44 (60.3%)	72.9 (19.2)		74.4 (23.4)		1.56 (−4.42, 7.55)	0.600
ECOG 1–3	29 (39.7%)	63.9 (17.1)		71.6 (20.1)		7.62 (0.59, 14.66)	**0.035**
**Race/Ethnicity**			0.712		0.824		
White/Non-Hispanic	58 (90.6%)	68.7 (18.3)		72.3 (22.8)		3.65 (−1.59, 8.90)	0.168
Other	6 (9.4%)	65.6 (25.3)		70.1 (25.1)		4.46 (−15.47, 24.39)	0.590

SD, standard deviation, CI, confidence interval, PBT, proton beam therapy, GTR, gross total resection, NTR, near total resection, STR, subtotal resection, CSI, craniospinal irradiation, ECOG, Eastern Cooperative Oncology Group Performance Status Scale

^1^n (%), ^2^Two Sample t-test, ^3^Paired two sample t-test

At baseline, participants who received CSI reported lower total, physical and psychosocial scores (*p* < 0.05) for both PPR and CSR outcomes. Additionally, the receipt of chemotherapy was associated with worse HRQoL across all PPR summary scores (*p* < 0.05). However, no significant associations between chemotherapy and CSR outcomes were identified.

Hydrocephalus was associated with worse HRQoL across all three CSR summary scores at baseline (*p* < 0.05), but no association was observed with PPR outcomes. Participants with an ECOG performance status of 0 at baseline demonstrated significantly better PPR total and physical HRQoL during the first week of PBT, compared to those with an ECOG status of 1–3 (*p* < 0.05). No significant differences in HRQoL during PBT were observed for other demographic or treatment variables including age at PBT, sex, tumor location, surgical extent, median household income or race/ethnicity.

### RT dose to substructures and HRQoL

Associations between dose to brain substructures and PPR HRQoL summary scores at a median of five years post PBT were evaluated. Due to the reduced completion rate of CSR questionnaires, analyses focused on PPR outcomes.

Across the total cohort (Table 4), higher mean doses to the whole brain, supratentorial brain, hypothalamus, optic chiasm, pituitary and right thalamus were significantly correlated with worse total, physical and psychosocial HRQoL (*r*= −0.24 to −0.39), *p* < 0.04). Similar associations were observed for maximum doses to the hypothalamus, pituitary and optic chiasm (*r*= −0.3 to −0.35, *p* ≤ 0.011). Additionally, higher mean doses to the left thalamus, pineal gland and left hippocampus correlated with worse total and psychosocial HRQoL (*r*= −0.24 to −0.28, *p* < 0.05). Mean dose to the corpus callosum was significantly associated with worse total and physical HRQoL (−0.24 to −0.25, *p* < 0.05).

**Table 4:** Correlation between radiation dose to brain substructures and parent-proxy reported HRQoL outcomes

Structure	PBT Dose, Mean(SD)	Total			Psychosocial			Physical		
		*n*	*R*	*p*	*n*	*R*	*p*	*n*	*R*	*p*
**Mean dose**										
Brain (whole)	14 (10)	76	−0.27	**0.0164**	76	−0.29	**0.0108**	76	−0.24	**0.0396**
Brain (supratentorial)	13 (10)	76	−0.28	**0.0157**	76	−0.28	**0.014**	76	−0.25	**0.0297**
Brain (infratentorial)	22 (20)	76	−0.09	0.4456	76	−0.12	0.3148	76	−0.06	0.612
Brainstem	27 (19)	76	−0.15	0.2048	76	−0.17	0.133	76	−0.11	0.3318
Corpus Callosum	16 (14)	71	−0.24	**0.0425**	71	−0.22	0.0716	71	−0.25	**0.0391**
Hippocampus (left)	20 (17)	72	−0.24	**0.0415**	72	−0.28	**0.0154**	72	−0.15	0.2025
Hippocampus (right)	20 (16)	74	−0.21	0.0773	74	−0.22	0.0574	74	−0.17	0.1448
Hypothalamus	20 (17)	70	−0.37	**0.0016**	70	−0.37	**0.0018**	70	−0.34	**0.0040**
Optic Chiasm	19 (18)	74	−0.39	**0.0007**	74	−0.37	**0.0012**	74	−0.38	**0.0008**
Pineal Gland	27 (22)	66	−0.25	**0.040**	66	−0.26	**0.0319**	66	−0.2	0.1035
Pituitary	18 (17)	71	−0.37	**0.0017**	71	−0.35	**0.0028**	71	−0.36	**0.0020**
Temporal Lobe (left)	15 (14)	75	−0.18	0.1231	75	−0.22	0.0553	75	−0.11	0.354
Temporal Lobe (right)	13 (12)	75	−0.17	0.1424	75	−0.18	0.1315	75	−0.15	0.1848
Thalamus (left)	21 (18)	75	−0.26	**0.0262**	75	−0.27	**0.0197**	75	−0.21	0.0761
Thalamus (right)	22 (18)	76	−0.26	**0.0257**	76	−0.24	**0.0344**	76	−0.25	**0.0268**
**Maximum dose**										
Brain (whole)	54 (7)	76	0.1	0.3993	76	0.11	0.3346	76	0.07	0.5311
Brain (supratentorial)	53 (7)	76	0.02	0.8711	76	0.03	0.8078	76	0.01	0.9364
Brain (infratentorial)	45 (18)	76	0.01	0.9108	76	0.0	0.9934	76	0.02	0.8431
Brainstem	43 (18)	76	0.0	0.9699	76	−0.01	0.9125	76	0.04	0.7566
Corpus Callosum	35 (20)	71	−0.12	0.2993	71	−0.1	0.4198	71	−0.14	0.2331
Hippocampus (left)	33 (21)	72	−0.21	0.0736	72	−0.25	**0.0371**	72	−0.14	0.2474
Hippocampus (right)	35 (20)	74	−0.19	0.1129	74	−0.18	0.1212	74	−0.18	0.1162
Hypothalamus	29 (19)	70	−0.32	**0.0062**	70	−0.31	**0.0087**	70	−0.3	**0.0111**
Optic Chiasm	23 (19)	74	−0.32	**0.0048**	74	−0.31	**0.0073**	74	−0.32	**0.0048**
Pineal Gland	30 (23)	66	−0.24	0.0557	66	−0.25	**0.0444**	66	−0.18	0.1425
Pituitary	22 (18)	71	−0.35	**0.0024**	71	−0.34	**0.0038**	71	−0.35	**0.0025**
Temporal Lobe (left)	41 (20)	75	−0.19	0.1066	75	−0.23	0.0509	75	−0.12	0.3055
Temporal Lobe (right)	42 (19)	75	−0.15	0.2089	75	−0.11	0.3299	75	−0.18	0.1137
Thalamus (left)	36 (21)	75	−0.27	**0.0203**	75	−0.27	**0.0187**	75	−0.23	**0.0468**
Thalamus (right)	36 (22)	76	−0.22	0.0544	76	−0.22	0.062	76	−0.21	0.0703

p<0.05 are shown in bold

Subgroup analyses (Table 5) were performed comparing children who received CSI with those who received focal PBT. The strongest correlations were observed among children who received CSI. Significant correlations were observed between mean dose to the whole brain, left and right hippocampi, and worse HRQoL across all three summary scores (*r*= −0.51 to −0.74, *p* < 0.05). The strongest correlations were between mean hippocampal dose and physical health outcomes (left hippocampus: *r* = −0.74, right hippocampus: *r* = −0.73, *p* < 0.001), as well as between the left hippocampus and total HRQoL (*r* = −0.74, *p* = 0.0007). Higher maximum doses to the hippocampi were also correlated with lower physical health scores (*p* < 0.05). Increased mean and maximum doses to the hypothalamus and optic chiasm were significantly associated with lower total and physical health scores (*r* = −0.51 to −0.79, *p* < 0.05). Higher maximum doses to the pituitary were also correlated with worse total, physical and psychosocial HRQoL outcomes (*r* = −0.52 to −0.77, *p* < 0.05). Correlations between RT dose and poorer physical health outcomes were also identified for other structures, including the corpus callosum, supratentorial brain, temporal lobes and thalami (*r* = −0.51 to −0.68, *p* < 0.05).

**Table 5:** Correlation between radiation dose to brain substructures and parent-proxy reported HRQoL outcomes in children, stratified by field extent

Structure	CSI (*n* = 17)								Focal PBT (*n* = 59)							
	PBT Dose, Mean (SD)	n	Total		Physical		Psychosocial		PBT Dose, Mean (SD)	n	Total		Physical		Psychosocial	
			R	p	R	p	R	p			R	p	R	p	R	p
**Mean dose**																
Brain (whole)	31(4)	17	−0.55	**0.022**	−0.68	**0.0025**	−0.51	**0.0386**	9(5)	59	−0.22	0.0991	−0.17	0.1955	−0.24	0.0729
Brain (supratentorial)	29(5)	17	−0.46	0.0602	−0.65	**0.0048**	−0.4	0.1155	9(6)	59	−0.23	0.0838	−0.2	0.1344	−0.23	0.0838
Brain (infratentorial)	47(7)	17	−0.44	0.0779	−0.39	0.1183	−0.42	0.097	14(16)	59	0.05	0.6869	0.07	0.622	0.02	0.8752
Brainstem	49(5)	17	−0.22	0.4024	−0.13	0.6129	−0.22	0.3997	20(17)	59	−0.04	0.7444	−0.03	0.7978	−0.07	0.5828
Corpus Callosum	30(8)	17	−0.4	0.1143	−0.65	**0.0048**	−0.31	0.2256	11(13)	54	−0.19	0.1784	−0.17	0.2075	−0.15	0.2848
Hippocampus (left)	40(6)	17	−0.74	**0.0007**	−0.74	**0.0006**	−0.68	**0.0028**	13(14)	55	−0.14	0.3077	−0.04	0.7855	−0.19	0.1599
Hippocampus (right)	38(6)	17	−0.66	**0.004**	−0.73	**0.0009**	−0.57	**0.0171**	15(14)	57	−0.12	0.3616	−0.1	0.4564	−0.13	0.3414
Hypothalamus	33(8)	17	−0.56	**0.0183**	−0.7	**0.0019**	−0.45	0.0708	16(18)	53	−0.29	**0.0329**	−0.28	**0.0455**	−0.29	**0.0353**
Optic Chiasm	27(5)	17	−0.52	**0.0334**	−0.71	**0.0014**	−0.44	0.0807	17(20)	57	−0.35	**0.0079**	−0.36	**0.0067**	−0.33	**0.0123**
Pineal Gland	50(4)	14	−0.01	0.9702	−0.11	0.6963	0.12	0.6914	20(21)	52	−0.26	0.0656	−0.2	0.1511	−0.27	**0.0495**
Pituitary	28(5)	17	−0.47	0.0572	−0.68	**0.0029**	−0.36	0.1507	14(18)	54	−0.3	**0.0298**	−0.3	**0.0267**	−0.28	**0.0406**
Temporal Lobe (left)	31(4)	17	−0.43	0.0817	−0.61	**0.0097**	−0.36	0.1582	10(12)	58	−0.1	0.4413	−0.01	0.9215	−0.16	0.2165
Temporal Lobe (right)	30(4)	16	−0.42	0.1099	−0.59	**0.0165**	−0.34	0.1915	9(9)	59	−0.12	0.3717	−0.13	0.3412	−0.11	0.3988
Thalamus (left)	37(10)	17	−0.43	0.0856	−0.65	**0.0044**	−0.31	0.2314	16(18)	58	−0.23	0.0847	−0.16	0.2366	−0.25	0.0614
Thalamus (right)	36(10)	17	−0.42	0.0907	−0.64	**0.0061**	−0.31	0.2275	18(18)	59	−0.22	0.0924	−0.2	0.1257	−0.22	0.1013
**Maximum dose**																
Brain (whole)	55(1)	17	−0.04	0.8701	−0.17	0.5213	0.06	0.8297	53(8)	59	0.12	0.3614	0.12	0.3794	0.13	0.3268
Brain (supratentorial)	55(1)	17	−0.37	0.1477	−0.44	0.0809	−0.29	0.2663	53(8)	59	0.05	0.6967	0.06	0.6537	0.06	0.6356
Brain (infratentorial)	55(1)	17	0.05	0.8517	−0.05	0.8624	0.11	0.6836	42(19)	59	0.08	0.5373	0.1	0.4321	0.06	0.6366
Brainstem	54(0)	17	0.05	0.8516	0.14	0.5997	−0.03	0.9144	40(19)	59	0.04	0.7814	0.06	0.673	0.02	0.8684
Corpus Callosum	42(11)	17	−0.29	0.2557	−0.51	**0.0354**	−0.19	0.4679	32(21)	54	−0.1	0.4854	−0.07	0.6248	−0.07	0.6186
Hippocampus (left)	53(3)	17	−0.4	0.1155	−0.5	**0.0426**	−0.29	0.2557	27(20)	55	−0.15	0.2735	−0.06	0.6563	−0.19	0.1664
Hippocampus (right)	52(4)	17	−0.43	0.0886	−0.52	**0.031**	−0.28	0.2706	31(21)	57	−0.12	0.3745	−0.11	0.4178	−0.11	0.4009
Hypothalamus	41(10)	17	−0.51	**0.038**	−0.55	**0.0216**	−0.39	0.1231	25(20)	53	−0.27	0.0535	−0.25	0.0757	−0.26	0.065
Optic Chiasm	30(6)	17	−0.63	**0.0063**	−0.79	**0.0002**	−0.54	**0.0258**	21(20)	57	−0.31	**0.0183**	−0.32	**0.0163**	−0.29	**0.0266**
Pineal Gland	53(2)	14	−0.1	0.7363	−0.19	0.5112	0.05	0.8636	24(22)	52	−0.24	0.0848	−0.18	0.2049	−0.26	0.0613
Pituitary	30(6)	17	−0.61	**0.0098**	−0.77	**0.0003**	−0.52	**0.0324**	19(20)	54	−0.29	**0.0329**	−0.3	**0.0252**	−0.27	**0.0455**
Temporal Lobe (left)	54(1)	17	−0.44	0.0798	−0.57	**0.0165**	−0.32	0.2104	37(21)	58	−0.13	0.3245	−0.05	0.7201	−0.17	0.1985
Temporal Lobe (right)	54(1)	16	−0.3	0.2509	−0.41	0.1166	−0.25	0.36	38(20)	59	−0.08	0.5529	−0.12	0.3577	−0.04	0.7486
Thalamus (left)	52(4)	17	−0.35	0.1628	−0.51	**0.0349**	−0.22	0.4054	31(22)	58	−0.24	0.0728	−0.17	0.1936	−0.25	0.0606
Thalamus (right)	51(3)	17	−0.22	0.4	−0.36	0.1541	−0.1	0.708	31(23)	59	−0.19	0.1462	−0.16	0.2315	−0.19	0.1479

p<0.05 are shown in bold

In children who did not receive CSI, correlations were weaker but remained significant. Higher mean doses to the hypothalamus, optic chiasm and pituitary were correlated with worse total, physical and psychosocial health outcomes (*r* = −0.28 to −0.35, *p* < 0.05). Associations between maximum dose and HRQoL were also observed for these structures, with the correlations for optic chiasm and pituitary reaching statistical significance.

No significant relationships were observed between RT dose to the infratentorial brain or brainstem and HRQoL.

## Discussion

Enhancing knowledge of radiation tolerance and refining dose constraints has become a major focus of international pediatric radiation oncology research efforts [31]. To our knowledge, this is the first study to analyze the relationship between PBT dose to three-dimensionally contoured brain substructures and HRQoL outcomes in pediatric brain tumor survivors. At a median follow up of five years post PBT, our findings demonstrate that higher doses to brain substructures are associated with poorer HRQoL, and we have identified key substructures where dose sparing may improve outcomes.

Across the entire cohort, higher mean doses to the whole brain, supratentorial brain, corpus callosum, left hippocampus, hypothalamus, optic chiasm, pineal gland, pituitary and thalamus were significantly correlated with worse HRQoL. When stratified by treatment extent, we observed moderate to strong correlations in children who received CSI, particularly between higher doses to these structures and worse physical HRQoL. In contrast, weak associations were identified in children treated with focal PBT, with significant correlations observed for the hypothalamus, optic chiasm and pituitary. The differing correlation strength supports a potential dose-volume effect, as children receiving CSI received higher radiation doses to these structures.

Our findings support the growing role of substructure-informed planning in pediatric RT. Based on observed associations between dose to multiple brain substructures and worse HRQoL, we recommend that key regions such as the hippocampi, hypothalamus, pituitary, thalamus and corpus callosum should be routinely contoured and considered, with RT dose reduced where feasible. This aligns with European Particle Therapy Network (EPTN) contouring atlas, recommending the segmentation of non-traditional organs at risk, such as the thalamus and corpus callosum [22]. As correlations were observed with mean doses, our study reiterates the importance of achieving as low dose as possible to the brain, as radiation doses well below defined dose constraints may impact the HRQoL of survivors.

Our results build on prior work linking regional brain irradiation to long-term HRQoL deficits. A previous study of childhood cancer survivors treated with photon RT from 1970 to 1986 identified associations between irradiation of the temporal region and impaired physical health, social functioning and emotional functioning HRQoL domains [16]. As the analysis was based on 2D-dose estimates derived from field sizes, our study provides updated insights with modern CT-based planning, dosimetric evaluation and outcomes of patients treated with PBT. Our associations between hippocampi dose and lower total HRQoL, physical health and psychosocial health, and associations between temporal lobes and physical health in patients treated with CSI, support prior temporal region observations. These findings also align with the growing interest in hippocampal-sparing techniques. Recent studies have explored the feasibility of hippocampal-sparing CSI for children with medulloblastoma [32–34], following trials showing that hippocampal-sparing can reduce memory-related cognitive deficits in adults with brain metastases [35–37]. Our results support the rationale for substructure-informed planning techniques such as hippocampal-sparing CSI, and suggest that preservation of structures crucial for neurocognitive functioning may improve HRQoL outcomes as well.

Our findings also reflect data from adult brain tumor survivors. A study of patients treated with conformal RT, intensity-modulated RT and PBT also observed an association between higher mean doses to the whole brain, hippocampi, temporal lobes and thalamus, and lower HRQoL scores at a similar length of follow-up (4.6 years) [15]. Although statistical methods and patient populations differed, the emerging evidence from brain cancer patients treated with conventional RT protocols suggests that minimizing exposure to brain substructures may preserve HRQoL for survivors.

The associations between dose to the hypothalamus, pituitary and optic chiasm, and worse HRQoL outcomes in the total cohort, CSI and focal PBT groups are also noteworthy. These findings may be indicative of central endocrine toxicity resulting from hypothalamic-pituitary axis irradiation. Alternatively, they could relate to other effects not yet understood, such as how these structures may mediate fatigue, emotional regulation and cognitive functioning. Additionally, based on the relationship between RT dose and neuropsychological functioning decline [38–40], we included corpus callosum in the analysis. We found a weak association between corpus callosum dose and total and physical HRQoL across the entire cohort, and a moderate association with physical HRQoL in the CSI subgroup. These findings further highlight the need for careful dose consideration to midline structures during RT planning.

Interestingly, although prior adult studies have found associations between higher mean dose to the brainstem and patient-reported fatigue or lower HRQoL [15, 41], we did not observe significant brainstem-related correlations in our pediatric cohort. This discrepancy may reflect differences in tumor characteristics (e.g. a greater predominance of posterior fossa tumors in our cohort), treatment protocols, follow-up duration and/or PROM instruments.

Our findings also highlight the impact of treatment intensity on HRQoL outcomes. Not surprisingly, both CSI and chemotherapy were negatively correlated with HRQoL outcomes during the first week of PBT, consistent with outcomes of a prior cohort treated with PBT [42]. This likely reflects the acute side effects associated with these treatments. While these correlations did not reach significance at follow-up in the univariate analyses, the strongest associations between substructure dose and HRQoL at follow-up were observed in patients who received CSI. This suggests that the higher doses delivered with CSI, along with factors such as greater field extent, the cumulative burden of multimodal therapy and underlying diagnostic differences, may contribute to worse HRQoL. However, the small sample size of the CSI subgroup limits the generalizability of our findings. Our cohort also had lower HRQoL than population norms at baseline, with the great differences observed in school and physical functioning domains. This likely reflects the impact of disease and pre-PBT treatment on physical abilities, cognition and school attendance. Assessment and clinical review of HRQoL outcomes during treatment may also provide additional information to further support patients.

Our study has several strengths, including prospective HRQoL data collection from children treated with modern RT techniques and leveraging detailed dosimetric analyses from multiple centers. However, there are some limitations. The sample size restricted our ability to evaluate potential confounding factors such as diagnosis. Potential differences in HRQoL based on tumor location were explored, and grouping patients by CSI vs. focal PBT allowed us to partially account for variations in diagnosis and treatment intensity. However, this approach does not capture the influence of tumor biology or other treatment modalities on HRQoL outcomes. Additionally, while we explored the impact of race, ethnicity, and socioeconomic status, the paucity of non-white participants limited our ability to adequately assess these factors. Socioeconomic status was estimated using median household income by zip code, which may not accurately reflect individual circumstances.

Furthermore, comprehensive toxicity data was not available, limiting our ability to evaluate how late occurring side-effects may influence HRQoL. Correlations identified for one structure, such as the optic chiasm, may serve as a surrogate for adjacent structures, given its proximity to the hypothalamic-pituitary axis and medial temporal lobes. It remains difficult to speculate on the specific impact of chiasmal dose on HRQoL when vision remains intact. Future research should include comprehensive assessment combining HRQoL outcomes, clinical and toxicity data to assess the complex relationships between diagnosis, radiation dose, toxicity and HRQoL in this population. Importantly, HRQoL should continue to be measured and evaluated into extended follow-up, to support investigation of long-term impacts of substructure dose and late effects.

## Conclusion

Modern RT techniques, such as PBT and substructure-informed planning, offer opportunities to minimize dose to critical structures while maintaining treatment efficacy. Our findings provide new insights into the relationship between radiation dose and HRQoL outcomes in children with brain tumors. Higher doses to the whole brain, supratentorial brain, hippocampi, hypothalamus, pituitary, thalamus and corpus callosum were associated with poorer HRQoL post-PBT. Substructure-informed planning techniques to reduce RT dose where feasible may enhance HRQoL for pediatric brain tumor survivors.

## Supplementary Information

Below is the link to the electronic supplementary material.

Supplementary material 1 (DOCX 88.1 kb)

## Data Availability

The data that support the findings of this study are available from the corresponding author upon reasonable request.
