# Supplementary material for: Impact of substructure radiation dose on health-related quality of life in children with brain tumors: a Pediatric Proton/Photon Consortium Registry (PPCR) study
Source: J Neurooncol. 2025 Sep 9;175(3):1443–53. doi: 10.1007/s11060-025-05211-w (PMC12511131; doi:10.1007/s11060-025-05211-w)
Supplement: Supplementary file 1 — Supplementary material 1 (DOCX 88.1 kb) [file 11060_2025_5211_MOESM1_ESM.docx]

**Supplementary Material – Journal of Neuro Oncology**

**Impact of substructure radiation dose on health-related quality of life in children with brain tumors: a Pediatric Proton/Photon Consortium Registry (PPCR) study**
Mikaela Doig, BMRS(Hons)^1,2,3^, Jae Lee, MD, PhD^4^, Young Kwok, MD^5^, Iain MacEwan, MD^6^, Suzanne Wolden, MD^7^, Keith Allison, MS^8^, Sara Dennehy, MS^8^, Benjamin Bajaj, MA^8^, Michala Short, PhD^1^, Peter Gorayski, FRANZCR^1,2,3^, Eva Bezak, PhD^1^, Torunn I. Yock, MD, MCH^8,9^

^1^Allied Health and Human Performance Academic Unit, University of South Australia, Adelaide, Australia. Email: Mikaela.Doig@unisa.edu.au

^2^ Department of Radiation Oncology, Royal Adelaide Hospital, Adelaide, Australia

^3^ Australian Bragg Centre for Proton Therapy and Research, Adelaide, Australia
^4^ Department of Radiation Oncology, ProCure Proton Therapy Center, Somerset, USA

^5^ Department of Radiation Oncology, Maryland Proton Treatment Center, University of Maryland, Baltimore, USA

^6^ Department of Radiation Oncology, California Protons Cancer Therapy Center, San Diego, USA

^7^ Department of Radiation Oncology, New York Proton Center, New York, USA

^8^ Department of Radiation Oncology, Massachusetts General Hospital, Boston, USA

^9^ Harvard Medical School, Harvard University, Boston, USA

# Table S1. Yearly changes in health-related quality of life

| Parent/Child | Score | n | Baseline | Yearly Change (95% CI) | p |
| --- | --- | --- | --- | --- | --- |
| Parent | Total Core | 76 | 69.1 (18.8) | 1.2 (0.4 to 2.0) | **0.0031** |
|  | Psychosocial | 76 | 71.4 (16.3) | 0.5 (-0.3 to 1.2) | 0.2029 |
|  | Physical | 76 | 65.6 (27.3) | 2.4 (1.2 to 3.6) | **0.00010** |
|  | Emotional | 76 | 68.7 (19.0) | 1.0 (0.1 to 1.8) | **0.0283** |
|  | Social | 76 | 79.9 (15.8) | -0.4 (-1.3 to 0.4) | 0.3429 |
|  | School | 43 | 61.0 (23.7) | 1.9 (0.6 to 3.2) | **0.0043** |
| Child | Total Core | 49 | 75.8 (16.1) | 0.3 (-0.5 to 1.1) | 0.4485 |
|  | Psychosocial | 48 | 77.2 (14.1) | 0.0 (-0.8 to 0.9) | 0.9855 |
|  | Physical | 50 | 74.5 (23.4) | 0.7 (-0.3 to 1.7) | 0.1801 |
|  | Emotional | 49 | 75.4 (18.1) | 0.3 (-0.7 to 1.3) | 0.5923 |
|  | Social | 48 | 85.4 (15.7) | -0.8 (-1.8 to 0.1) | 0.0915 |
|  | School | 38 | 67.6 (18.9) | 0.9 (-0.4 to 2.1) | 0.1662 |

#

### Table S2. Univariate analysis of parent-proxy reported psychosocial summary score and clinical variables

|  |  | **Baseline** | | **Last follow-up** | |  |  |
| --- | --- | --- | --- | --- | --- | --- | --- |
| **Psychosocial** | **n = 76**^1^ | **Mean (SD)** | **p-value**^2^ | **Mean (SD)** | **p-value**^2^ | **Mean difference (95% CI)** | **p-value**^3^ |
| **Overall** |  | 71.4, (16.3) |  | 73.2, (20.0) |  | 1.77, (-2.27, 5.81) | 0.384 |
| **Age at PBT** |  |  | 0.154 |  | 0.810 |  |  |
| ≤ 7 years-old | 33 (43.4%) | 74.5, (13.7) |  | 73.8, (19.1) |  | -0.65, (-6.44, 5.14) | 0.821 |
| > 7 years-old | 43 (56.6%) | 69.1, (17.9) |  | 72.7, (20.9) |  | 3.63, (-2.1, 9.36) | 0.208 |
| **Sex** |  |  | 0.877 |  | 0.777 |  |  |
| Male | 37 (48.7%) | 71.1, (15.8) |  | 73.9, (19.8) |  | 2.75, (-2.96, 8.46) | 0.335 |
| Female | 39 (51.3%) | 71.7, (17.0) |  | 72.6, (20.5) |  | 0.85, (-5.1, 6.8) | 0.775 |
| **Tumor location** |  |  | 0.780 |  | 0.724 |  |  |
| Supratentorial | 45 (59.2%) | 71.0, (15.2) |  | 73.9, (20.3) |  | 2.89, (-2.67, 8.45) | 0.300 |
| Infratentorial | 31 (40.8%) | 72.1, (18.0) |  | 72.2, (19.9) |  | 0.15, (-5.92, 6.23) | 0.960 |
| **Surgical Extent** |  |  | 0.564 |  | 0.729 |  |  |
| GTR/ NTR | 51 (72.9%) | 73.0, (16.6) |  | 73.9, (19.2) |  | 0.94, (-3.54, 5.42) | 0.676 |
| STR | 19 (27.1%) | 70.4, (15.9) |  | 72.0, (23.3) |  | 1.6, (-8.36, 11.55) | 0.740 |
| **Chemotherapy** |  |  | **0.047** |  | 0.052 |  |  |
| Chemotherapy | 47 (61.8%) | 68.5, (16.0) |  | 69.7, (20.4) |  | 1.19, (-4.38, 6.77) | 0.669 |
| No chemotherapy | 29 (38.2%) | 76.1, (16.0) |  | 78.9, (18.4) |  | 2.72, (-3.21, 8.64) | 0.356 |
| **Hydrocephalus** |  |  | 0.321 |  | 0.115 |  |  |
| Hydrocephalus | 33 (46.5%) | 69.1, (17.0) |  | 69.3, (21.7) |  | 0.23, (-6.66, 7.12) | 0.946 |
| No hydrocephalus | 38 (53.5%) | 73.0, (16.0) |  | 77.0, (18.7) |  | 3.96, (-1.42, 9.35) | 0.145 |
| **Craniospinal irradiation** |  |  | **0.042** |  | 0.224 |  |  |
| CSI dose | 17 (22.4%) | 64.4, (16.4) |  | 68.0, (17.8) |  | 3.61, (-6.25, 13.46) | 0.449 |
| No CSI dose | 59 (77.6%) | 73.5, (15.9) |  | 74.7, (20.5) |  | 1.25, (-3.28, 5.77) | 0.583 |
| **Median income of zipcode** |  |  | 0.071 |  | 0.193 |  |  |
| ≤ $95,000 | 32 (46.4%) | 76.2, (15.9) |  | 75.3, (20.4) |  | -0.9, (-6.28, 4.48) | 0.734 |
| > $95,000 | 37 (53.6%) | 69.1, (16.2) |  | 68.8, (20.3) |  | -0.26, (-6.04, 5.53) | 0.928 |
| **ECOG** |  |  | 0.222 |  | 0.650 |  |  |
| ECOG 0 | 44 (60.3%) | 73.6, (17.3) |  | 74.0, (21.3) |  | 0.31, (-4.82, 5.44) | 0.903 |
| ECOG 1-3 | 29 (39.7%) | 68.9, (14.2) |  | 71.8, (18.2) |  | 2.88, (-3.73, 9.5) | 0.380 |
| **Race/Ethnicity** |  |  | 0.358 |  | 0.603 |  |  |
| White/Non-Hispanic | 58 (90.6%) | 70.2, (16.0) |  | 72.4, (20.3) |  | 2.18, (-2.43, 6.78) | 0.348 |
| Other | 6 (9.4%) | 76.7, (18.4) |  | 67.8, (23.8) |  | -8.87, (-26.08, 8.34) | 0.242 |
| ^1^n (%),^2^Two Sample t-test, ^3^Paired two sample t-test  Abbreviations: SD, standard deviation, CI, confidence interval, PBT, proton beam therapy, GTR, gross total resection, NTR, near total resection, STR, subtotal resection, CSI, craniospinal irradiation, ECOG, Eastern Cooperative Oncology Group Performance Status Scale | | | | | | | |

###

### Table S3. Univariate analysis of parent-proxy reported physical summary score and clinical variables

|  |  | **Baseline** | | **Last follow-up** | |  |  |
| --- | --- | --- | --- | --- | --- | --- | --- |
| **Physical** | **n = 76**^1^ | **Mean (SD)** | **p-value**^2^ | **Mean (SD)** | **p-value**^2^ | **Mean difference (95% CI)** | **p-value**^3^ |
| **Overall** |  | 65.6, (27.3) |  | 73.7, (28.2) |  | 8.1, (1.35, 14.85) | **0.019** |
| **Age at PBT** |  |  | 0.421 |  | 0.783 |  |  |
| ≤ 7 years-old | 33 (43.4%) | 68.5, (24.4) |  | 74.7, (27.2) |  | 6.23, (-5.95, 18.42) | 0.305 |
| > 7 years-old | 43 (56.6%) | 63.4, (29.3) |  | 72.9, (29.2) |  | 9.53, (1.67, 17.4) | **0.019** |
| **Sex** |  |  | 0.708 |  | 0.534 |  |  |
| Male | 37 (48.7%) | 66.8, (25.5) |  | 75.8, (27.1) |  | 8.97, (0.41, 17.53) | **0.040** |
| Female | 39 (51.3%) | 64.4, (29.1) |  | 71.7, (29.4) |  | 7.28, (-3.45, 18) | 0.178 |
| **Tumor location** |  |  | 0.164 |  | 0.994 |  |  |
| Supratentorial | 45 (59.2%) | 69.2, (24.7) |  | 73.7, (29.7) |  | 4.46, (-4.61, 13.52) | 0.327 |
| Infratentorial | 31 (40.8%) | 60.3, (30.2) |  | 73.7, (26.3) |  | 13.39, (3.04, 23.74) | **0.013** |
| **Surgical Extent** |  |  | 0.582 |  | 0.386 |  |  |
| GTR/ NTR | 51 (72.9%) | 67.3, (27.1) |  | 75.5, (26.1) |  | 8.19, (0.05, 16.32) | **0.049** |
| STR | 19 (27.1%) | 63.1, (30.4) |  | 68.8, (35.1) |  | 5.6, (-9.88, 21.08) | 0.457 |
| **Chemotherapy** |  |  | **0.048** |  | 0.175 |  |  |
| Chemotherapy | 47 (61.8%) | 60.8, (27.7) |  | 70.2, (30.8) |  | 9.48, (0.19, 18.77) | **0.046** |
| No chemotherapy | 29 (38.2%) | 73.4, (25.1) |  | 79.3, (22.7) |  | 5.87, (-4.07, 15.8) | 0.236 |
| **Hydrocephalus** |  |  | 0.157 |  | 0.288 |  |  |
| Hydrocephalus | 33 (46.5%) | 60.0, (27.9) |  | 70.7, (27.2) |  | 10.66, (-0.37, 21.69) | 0.058 |
| No hydrocephalus | 38 (53.5%) | 69.0, (25.2) |  | 77.6, (27.4) |  | 8.58, (-0.5, 17.67) | 0.063 |
| **Craniospinal irradiation** |  |  | **<0.001** |  | 0.574 |  |  |
| CSI dose | 17 (22.4%) | 45.7, (30.7) |  | 70.3, (24.0) |  | 24.53, (8.69, 40.37) | **0.005** |
| No CSI dose | 59 (77.6%) | 71.3, (23.5) |  | 74.7, (29.4) |  | 3.37, (-3.84, 10.57) | 0.353 |
| **Median income of zipcode** |  |  | 0.158 |  | 0.673 |  |  |
| ≤ $95,000 | 32 (46.4%) | 71.1, (26.2) |  | 73.4, (29.1) |  | 2.37, (-6.64, 11.37) | 0.595 |
| > $95,000 | 37 (53.6%) | 61.6, (28.6) |  | 70.5, (28.9) |  | 8.89, (-2.16, 19.94) | 0.111 |
| **ECOG** |  |  | **0.019** |  | 0.502 |  |  |
| ECOG 0 | 44 (60.3%) | 71.7, (26.2) |  | 75.7, (29.1) |  | 3.98, (-4.56, 12.51) | 0.353 |
| ECOG 1-3 | 29 (39.7%) | 56.4, (27.5) |  | 71.2, (26.7) |  | 14.77, (3.14, 26.39) | **0.015** |
| **Race/Ethnicity** |  |  | 0.290 |  | 0.868 |  |  |
| White/Non-Hispanic | 58 (90.6%) | 66.0, (26.8) |  | 72.4, (29.6) |  | 6.37, (-1.38, 14.12) | 0.105 |
| Other | 6 (9.4%) | 53.1, (40.6) |  | 74.5, (28.4) |  | 21.35, (-13.24, 55.95) | 0.173 |
| ^1^n (%), ^2^Two Sample t-test, ^3^Paired two sample t-test  Abbreviations: SD, standard deviation, CI, confidence interval, PBT, proton beam therapy, GTR, gross total resection, NTR, near total resection, STR, subtotal resection, CSI, craniospinal irradiation, ECOG, Eastern Cooperative Oncology Group Performance Status Scale | | | | | | | |

# Table S4. Univariate analysis of child-self reported total HRQoL and clinical variables

###

|  |  | **Baseline** | | **Last follow-up** | |  |  |
| --- | --- | --- | --- | --- | --- | --- | --- |
| **Total Core Score** | **n = 49**^1^ | **Mean (SD)** | **p-value**^2^ | **Mean (SD)** | **p-value**^2^ | **Mean difference (95% CI)** | **p-value**^3^ |
| **Overall** |  | 75.8, (16.1) |  | 80.0, (16.7) |  | 2.54, (-2.68, 7.76) | 0.333 |
| **Age at PBT** |  |  | 0.820 |  | 0.762 |  |  |
| ≤ 7 years-old | 10 (20.4%) | 76.8, (11.6) |  | 79.0, (17.4) |  | 2.22, (-12.53, 16.98) | 0.741 |
| > 7 years-old | 39 (79.6%) | 75.5, (17.1) |  | 76.8, (21.3) |  | 1.31, (-4.13, 6.75) | 0.629 |
| **Sex** |  |  | 0.896 |  | 0.065 |  |  |
| Male | 25 (51.0%) | 75.5, (15.5) |  | 82.5, (15.1) |  | 7.06, (0.6, 13.53) | **0.033** |
| Female | 24 (49.0%) | 76.1, (17.0) |  | 71.8, (24.0) |  | -4.31, (-11.71, 3.09) | 0.241 |
| **Tumor location** |  |  | 0.239 |  | 0.129 |  |  |
| Supratentorial | 33 (67.3%) | 77.6, (16.1) |  | 80.3, (19.1) |  | 2.69, (-2.26, 7.64) | 0.276 |
| Infratentorial | 16 (32.7%) | 71.8, (15.7) |  | 70.9, (22.2) |  | -0.98, (-13.4, 11.45) | 0.869 |
| **Surgical Extent** |  |  | 0.397 |  | 0.372 |  |  |
| GTR/ NTR | 28 (63.6%) | 78.3, (16.2) |  | 78.9, (20.3) |  | 0.57, (-5.44, 6.59) | 0.846 |
| STR | 16 (36.4%) | 74.1, (15.5) |  | 72.8, (23.3) |  | -1.21, (-12.21, 9.78) | 0.817 |
| **Chemotherapy** |  |  | 0.157 |  | 0.394 |  |  |
| Chemotherapy | 32 (65.3%) | 73.4, (14.9) |  | 75.4, (20.8) |  | 2.04, (-4.9, 8.98) | 0.553 |
| No chemotherapy | 17 (34.7%) | 80.2, (17.7) |  | 80.7, (19.9) |  | 0.46, (-6.6, 7.53) | 0.891 |
| **Hydrocephalus** |  |  | **0.003** |  | 0.243 |  |  |
| Hydrocephalus | 20 (44.4%) | 67.8, (13.3) |  | 72.7, (19.6) |  | 4.83, (-4.16, 13.82) | 0.275 |
| No hydrocephalus | 25 (55.6%) | 81.8, (16.0) |  | 80.1, (22.0) |  | -1.69, (-8.6, 5.22) | 0.618 |
| **Craniospinal irradiation** |  |  | **0.006** |  | 0.264 |  |  |
| CSI dose | 14 (28.6%) | 66.0, (12.1) |  | 72.0, (16.5) |  | 6, (-6.05, 18.06) | 0.302 |
| No CSI dose | 35 (71.4%) | 79.6, (15.9) |  | 79.3, (21.7) |  | -0.31, (-5.73, 5.11) | 0.909 |
| **Median income of zipcode** |  |  | 0.330 |  | 0.886 |  |  |
| ≤ $95,000 | 19 (43.2%) | 79.3, (13.9) |  | 76.8, (20.2) |  | -2.43, (-11.61, 6.75) | 0.585 |
| > $95,000 | 25 (56.8%) | 74.4, (17.6) |  | 75.9, (22.2) |  | 1.46, (-5.09, 8.02) | 0.649 |
| **ECOG** |  |  | 0.078 |  | 0.971 |  |  |
| ECOG 0 | 31 (67.4%) | 78.4, (15.7) |  | 76.3, (23.1) |  | -2.1, (-8.34, 4.13) | 0.496 |
| ECOG 1-3 | 15 (32.6%) | 69.7, (14.6) |  | 76.6, (15.3) |  | 6.88, (-2.78, 16.53) | 0.149 |
| **Race/Ethnicity** |  |  | 0.910 |  | 0.593 |  |  |
| White/Non-Hispanic | 37 (90.2%) | 76.4, (16.7) |  | 76.3, (22.2) |  | -0.1, (-6.26, 6.05) | 0.973 |
| Other | 4 (9.8%) | 75.4, (13.8) |  | 70.1, (16.8) |  | -5.31, (-26.54, 15.92) | 0.484 |
| ^1^n (%),^2^Two Sample t-test, ^3^Paired two sample t-test  Abbreviations: SD, standard deviation, CI, confidence interval, PBT, proton beam therapy, GTR, gross total resection, NTR, near total resection, STR, subtotal resection, CSI, craniospinal irradiation, ECOG, Eastern Cooperative Oncology Group Performance Status Scale | | | | | | | |

#

# Table S5. Univariate analysis of child-self reported psychosocial summary score and clinical variables

###

|  | **Baseline Last Follow up** | | | | | | |
| --- | --- | --- | --- | --- | --- | --- | --- |
| **Psychosocial** | **n = 49**^1^ | **Mean (SD)** | **p-value**^2^ | **Mean (SD)** | **p-value**^2^ | **Mean difference (95% CI)** | **p-value^3^** |
| **Overall** |  | 77.2, (14.1) |  | 79.1, (16.6) |  | 1.29, (-3.86, 6.45) | 0.615 |
| **Age at RT** |  |  | 0.693 |  | 0.847 |  |  |
| ≤ 7 years-old | 10 (20.4%) | 75.6, (8.1) |  | 77.7, (17.9) |  | 2.05, (-11.42, 15.52) | 0.739 |
| > 7 years-old | 39 (79.6%) | 77.6, (15.3) |  | 76.3, (20.1) |  | -1.1, (-6.82, 4.61) | 0.698 |
| **Sex** |  |  | 0.515 |  | **0.049** |  |  |
| Male | 25 (51.0%) | 75.9, (13.8) |  | 81.9, (14.8) |  | 6.01, (-0.22, 12.25) | 0.058 |
| Female | 24 (49.0%) | 78.6, (14.5) |  | 71.0, (22.4) |  | -7.47, (-15.07, 0.13) | 0.054 |
| **Tumor location** |  |  | 0.377 |  | 0.260 |  |  |
| Supratentorial | 33 (67.3%) | 78.4, (14.1) |  | 78.8, (19.1) |  | 0.37, (-5.04, 5.78) | 0.891 |
| Infratentorial | 16 (32.7%) | 74.5, (14.1) |  | 72.0, (20.3) |  | -2.24, (-14.56, 10.08) | 0.703 |
| **Surgical Extent** |  |  | 0.571 |  | 0.413 |  |  |
| GTR/ NTR | 28 (63.6%) | 79.1, (14.7) |  | 78.3, (19.8) |  | -0.47, (-7.03, 6.09) | 0.884 |
| STR | 16 (36.4%) | 76.6, (13.1) |  | 73.0, (21.7) |  | -3.6, (-14.36, 7.17) | 0.487 |
| **Chemotherapy** |  |  | 0.230 |  | 0.336 |  |  |
| Chemotherapy | 32 (65.3%) | 75.4, (12.6) |  | 74.6, (19.7) |  | -0.57, (-7.73, 6.59) | 0.871 |
| No chemotherapy | 17 (34.7%) | 80.5, (16.3) |  | 80.3, (19.2) |  | -0.21, (-7.27, 6.85) | 0.950 |
| **Hydrocephalus** |  |  | **0.002** |  | 0.253 |  |  |
| Hydrocephalus | 20 (44.4%) | 69.5, (12.0) |  | 72.3, (18.9) |  | 3.01, (-6.44, 12.47) | 0.511 |
| No hydrocephalus | 25 (55.6%) | 82.5, (14.0) |  | 79.3, (21.0) |  | -3.15, (-10.21, 3.91) | 0.367 |
| **Craniospinal irradiation** |  |  | **0.021** |  | 0.346 |  |  |
| CSI dose | 14 (28.6%) | 69.6, (11.0) |  | 72.4, (17.1) |  | 3.08, (-9.89, 16.04) | 0.614 |
| No CSI dose | 35 (71.4%) | 80.0, (14.2) |  | 78.3, (20.4) |  | -1.75, (-7.25, 3.74) | 0.521 |
| **Median income of zipcode** |  |  | 0.172 |  | 0.668 |  |  |
| ≤ $95,000 | 19 (43.2%) | 81.3, (12.6) |  | 77.4, (17.4) |  | -3.88, (-12.12, 4.36) | 0.335 |
| > $95,000 | 25 (56.8%) | 75.3, (15.1) |  | 74.7, (22.3) |  | -0.61, (-7.84, 6.62) | 0.864 |
| **ECOG** |  |  | 0.140 |  | 0.768 |  |  |
| ECOG 0 | 31 (67.4%) | 79.3, (13.8) |  | 75.1, (21.6) |  | -3.98, (-10.32, 2.37) | 0.210 |
| ECOG 1-3 | 15 (32.6%) | 72.8, (13.4) |  | 77.0, (15.7) |  | 4.18, (-5.96, 14.32) | 0.391 |
| **Race/Ethnicity** |  |  | 0.870 |  | 0.333 |  |  |
| White/Non-Hispanic | 37 (90.2%) | 77.7, (15.0) |  | 76.5, (20.9) |  | 1.25, (-4.66, 7.16) | 0.670 |
| Other | 4 (9.8%) | 79.0, (8.2) |  | 65.8, (16.0) |  | 13.15, (-17.11, 43.4) | 0.261 |
| ^1^n (%),^2^Two Sample t-test, ^3^Paired two sample t-test  Abbreviations: SD, standard deviation, CI, confidence interval, PBT, proton beam therapy, GTR, gross total resection, NTR, near total resection, STR, subtotal resection, CSI, craniospinal irradiation, ECOG, Eastern Cooperative Oncology Group Performance Status Scale | | | | | | | |

# Table S6. Univariate analysis of child-self reported physical summary score and clinical variables

|  |  | **Baseline** | | **Last follow-up** | |  |  |
| --- | --- | --- | --- | --- | --- | --- | --- |
| **Physical** | **n = 49**^1^ | **Mean (SD)** | **p-value**^2^ | **Mean (SD)** | **p-value**^2^ | **Mean difference (95% CI)** | **p-value**^3^ |
| **Overall** |  | 74.5, (23.4) |  | 81.9, (19.1) |  | 3.81, (-2.65, 10.28) | 0.241 |
| **Age at PBT** |  |  | 0.541 |  | 0.682 |  |  |
| ≤ 7 years-old | 10 (20.4%) | 78.2, (22.1) |  | 81.6, (18.3) |  | 3.32, (-15.09, 21.73) | 0.693 |
| > 7 years-old | 39 (79.6%) | 73.1, (23.9) |  | 78.0, (25.5) |  | 4.92, (-1.77, 11.62) | 0.145 |
| **Sex** |  |  | 0.824 |  | 0.152 |  |  |
| Male | 25 (51.0%) | 74.9, (23.1) |  | 83.6, (18.7) |  | 8.7, (0.35, 17.06) | **0.042** |
| Female | 24 (49.0%) | 73.4, (24.3) |  | 73.7, (28.1) |  | 0.32, (-9.13, 9.77) | 0.945 |
| **Tumor location** |  |  | 0.301 |  | 0.059 |  |  |
| Supratentorial | 33 (67.3%) | 76.6, (22.8) |  | 83.2, (21.4) |  | 6.65, (0.85, 12.46) | **0.026** |
| Infratentorial | 16 (32.7%) | 69.1, (24.6) |  | 69.5, (27.1) |  | 0.36, (-15.45, 16.17) | 0.962 |
| **Surgical Extent** |  |  | 0.305 |  | 0.403 |  |  |
| GTR/ NTR | 28 (63.6%) | 78.3, (23.1) |  | 80.1, (23.0) |  | 1.82, (-5.72, 9.36) | 0.624 |
| STR | 16 (36.4%) | 70.7, (23.8) |  | 73.4, (28.6) |  | 2.75, (-9.72, 15.23) | 0.645 |
| **Chemotherapy** |  |  | 0.238 |  | 0.574 |  |  |
| Chemotherapy | 32 (65.3%) | 71.2, (24.1) |  | 77.3, (25.0) |  | 6.07, (-2.46, 14.61) | 0.157 |
| No chemotherapy | 17 (34.7%) | 79.6, (21.9) |  | 81.4, (22.6) |  | 1.82, (-6.93, 10.57) | 0.666 |
| **Hydrocephalus** |  |  | **0.025** |  | 0.228 |  |  |
| Hydrocephalus | 20 (44.4%) | 65.6, (21.5) |  | 73.2, (22.7) |  | 7.65, (-3.46, 18.76) | 0.166 |
| No hydrocephalus | 25 (55.6%) | 80.9, (22.3) |  | 82.1, (25.3) |  | 1.26, (-7.32, 9.85) | 0.764 |
| **Craniospinal irradiation** |  |  | **0.017** |  | 0.177 |  |  |
| CSI dose | 14 (28.6%) | 61.7, (23.6) |  | 71.4, (18.6) |  | 9.67, (-4.93, 24.27) | 0.176 |
| No CSI dose | 35 (71.4%) | 79.1, (21.8) |  | 81.7, (25.5) |  | 2.57, (-4.25, 9.38) | 0.449 |
| **Median income of zipcode** |  |  | 0.711 |  | 0.834 |  |  |
| ≤ $95,000 | 19 (43.2%) | 76.1, (19.4) |  | 76.5, (26.2) |  | 0.4, (-10.84, 11.63) | 0.942 |
| > $95,000 | 25 (56.8%) | 73.4, (26.7) |  | 78.1, (24.2) |  | 4.71, (-3.73, 13.15) | 0.261 |
| **ECOG** |  |  | 0.080 |  | 0.677 |  |  |
| ECOG 0 | 31 (67.4%) | 78.0, (22.3) |  | 79.0, (27.4) |  | 1.02, (-6.71, 8.75) | 0.789 |
| ECOG 1-3 | 15 (32.6%) | 65.3, (23.3) |  | 75.8, (17.8) |  | 10.5, (-1.7, 22.7) | 0.086 |
| **Race/Ethnicity** |  |  | 0.903 |  | 0.895 |  |  |
| White/Non-Hispanic | 37 (90.2%) | 74.3, (24.1) |  | 76.3, (26.3) |  | 2.03, (-5.6, 9.66) | 0.593 |
| Other | 4 (9.8%) | 72.7, (24.5) |  | 78.1, (18.9) |  | 5.38, (-12.4, 23.16) | 0.407 |
| ^1^n (%),^2^Two Sample t-test, ^3^Paired two sample t-test  Abbreviations: SD, standard deviation, CI, confidence interval, PBT, proton beam therapy, GTR, gross total resection, NTR, near total resection, STR, subtotal resection, CSI, craniospinal irradiation, ECOG, Eastern Cooperative Oncology Group Performance Status Scale | | | | | | | |
